# What roles do male partners play in the mothering experiences of women living with mental illness? A qualitative secondary analysis

**DOI:** 10.1186/s12888-019-2209-1

**Published:** 2019-07-25

**Authors:** Emily Beard, Anne Honey, Nicola Hancock, Ruby Awram, Melissa Miceli, Rachel Mayes

**Affiliations:** The University of Sydney, 75 East Street, Lidcombe, NSW 2141 Australia

**Keywords:** Partners, Mothering, Social support, Fathers, Qualitative secondary analysis

## Abstract

**Background:**

Mothers who live with mental illness face diverse challenges. Research suggests that partner support or otherwise is likely to have a crucial influence on mothers’ abilities to manage these challenges, yet little is known about how this plays out. In this study, we aimed to explore the roles played by male partners in the mothering experiences of women living with mental illness.

**Methods:**

We conducted a qualitative secondary analysis using interview data collected from 18 participants in two previous qualitative studies. Both studies focused on the mothering experiences of women who lived with mental illness. In both studies, the importance of male partners was striking. The data were analyzed using constant comparative analysis.

**Results:**

The roles of partners in women’s experiences of mothering were multiple and dynamic, with each male partner playing a unique combination of roles. These included: facilitator; teammate; unfulfilled potential; distraction; dismantler, and threat to child. Roles were influenced by: mothers’ interpretations; partners' behaviors, characteristics and circumstances; the family’s living and custody arrangements; mothers’ active management strategies; and a range of external controls and supports.

**Conclusions:**

Health professionals need to consider the complex roles partners play. This crucial aspect of mothers’ social environments can be optimized by directly supporting and enabling partners themselves, and by supporting mothers to actively shape their partners’ roles.

## Background

Many women live with mental illness whilst caring for dependent children. While precise statistics are lacking, estimates from the United States suggest that 65% of women with a diagnosed mental illness are mothers [1]. In Australia it is estimated that around one in five children live with a parent with mental illness [2, 3]. These mothers report facing a plethora of challenges including: stigma; social isolation; reduced energy; guilt; concern for children’s wellbeing; feelings of inadequacy; fear of custody loss; and difficulty balancing their mental health needs with the needs of their child [4–6].

### Family support

Positive family support has consistently been found to assist mothers living with mental illness to manage these challenges and stay well [6–9]. Mothers have reported several kinds of family support as important to them including: childcare during periods of ill-health [7, 10]; active listening and emotional support [7, 8]; and provision of helpful information and advice [9]. Children’s fathers and women’s other intimate partners (hereafter referred to collectively as ‘partners’) are likely to be important and unique sources of support for mothers living with mental illness.

### The importance of support from partners

The importance of partner support is demonstrated in general population research [11–13]. While relative importance is rarely researched, some studies suggest that partner support may take precedence over other relationships in women’s close support networks [14–17]. For example, one study [16] concluded that social support from one’s spouse was the only type of social support that significantly impacted life satisfaction and psychological wellbeing. Studies have further suggested that partner support can be specifically important for mothers. For example, women who have a supportive partner are more likely to avoid symptoms of depression and anxiety after childbirth [18] and have reduced parenting stress throughout the child’s early years [19]. Even when fathers assume the traditional role of secondary caregiver, the support they provide is described by mothers as critical to their well-being and ability to cope with difficult situations [20].

### Partners of mothers living with mental illness

While the role of partner support in women’s experiences of mothering with mental illness has not been specifically investigated, the importance of partners has emerged in related research about the wider experiences of mothers who live with mental illness. Across these studies, partners and ex-partners were frequently mentioned in quotes and examples demonstrating the influence of social networks on mothering (eg. [9, 10, 21]). One study, for example, found that all participants were able to identify at least one form of positive partner support, and four of the five mothers in current relationships described their partners as people they were able to talk to and rely on in times of need [22]. Other studies reported that partner support played a significant role in mothers’ ability to manage their daily lives and parental responsibilities [21, 23]. Although partners are not the focus of these studies, they collectively suggest that the support of partners is likely to be critical and unique in the lives of mothers living with mental illness.

However, for many mothers who live with mental illness, partner support may be compromised. These mothers are more likely than other mothers to be living without partners [24, 25] a status associated with financial hardship, perceived lack of social support and parenting stress [26, 27]. Mothers living with mental illness have been found to be at increased risk of experiencing financial and housing insecurity, partially due to lack of financial support from a partner [28].

Partnered mothers who live with mental illness may be more likely than other mothers to experience difficult relationships. For example, the prevalence of partner violence has been reported to be higher among women living with mental illness [29], with as many as 50 % of women in mental health treatment settings reporting partner violence [30]. Further, in qualitative studies, mothers living with mental illness often report that their partners abuse substances or have their own psychiatric diagnoses [22, 31]. Mothers have described unsupportive relationships as exacerbating their illness and hindering their mothering role [10].

Despite the specific importance of partners, previous studies have not differentiated between informal supports such as partners, grandparents and other family members [21, 22, 32]. To the researchers’ knowledge, a single study to date has presented findings about the roles of partners in the lives of mothers living with mental illness separately from their other family members. This paper [31] reported that both mothers and caseworkers perceived partners to be either ‘resources’ supporting mothers or ‘hindrances’ who undermined both mothering and recovery. Further, mothers were sometimes dependent on undermining partners, for example, due to fear of losing custody to the ‘well’ partner. While helpful, this study has limitations in its ability to provide an in-depth understanding of the roles of partners. Not only was it conducted 19 years ago, the objective of the study was to explore family relationships more generally and thus the specific role of partners was presented quite briefly. Further, the study used focus groups which, while useful in eliciting opinions and observing group interactions [33], do not gather in-depth understandings of participants’ individual stories as effectively as individual interviews [34]. Lastly, the perspectives of mothers and caseworkers were analyzed together, limiting the study’s ability to depict mothers’ unique views.

Partners of women with severe mental illness have sometimes been labeled as bad, burdened or ill [35]. Yet this is a simplistic view of their roles, which are likely to be considerably more nuanced and complex. For example, Booth and Booth [36], in a study looking at the place of men in the lives of mothers with intellectual disabilities, challenged stereotypes of these men as exploitative or damaged and mothers as “used, abused and abandoned” by them. The study used secondary analysis of data from several studies to reveal that, in fact, partners played diverse and often positive roles in the women’s lives.

In summary, little is known about the specific support mothers who live with mental illness experience from partners. While the difficulties they experience with partners are often highlighted, a nuanced understanding of partners’ complex roles in these women’s mothering experiences is missing from the literature. Developing such an understanding is necessary for mental health workers and other professionals to support these mothers in the context of their daily lives and circumstances. To begin to address this gap, we aimed to explore the roles of partners in the mothering experiences of women living with mental illness. In this paper we answer the following research questions: 1) What are partners’ roles in the mothering experiences of women living with mental illness?; and 2) What shapes these roles? In answering these questions we examine the perspectives of mothers, as partners’ roles in mothers’ lives can only be understood in terms of mothers’ experiences.

## Methods

### Design

We conducted a secondary analysis using in-depth interview data from two qualitative studies conducted by the authors [9, 37]. Study 1 was a phenomenological study that explored mothers’ lived experiences of mothering after the removal of a child by child protection services. Study 2 used a grounded theory approach to explore how mothers successfully managed the dual demands of mothering and mental health recovery.

In analysis of both datasets, the roles of partners (including children’s fathers and mothers’ other intimate partners and ex-partners) were clearly important to mothers’ experiences of mothering. However, they were not the main foci of the studies. The magnitude and diversity of partners’ influences warranted a more focused analysis of the role of partners in the mothering experiences of these women.

Qualitative secondary analysis, the use of existing data generated for other purposes, has increasingly been recognized as having the potential to provide useful information while optimizing the value of data collected, particularly for participant groups who are difficult to access or for whom participation carries some risk of distress or burden [38].

### Sample

Data were drawn from interviews with 18 participants. Study 1 involved eight mothers living with mental illness who had experienced the removal of a child by child protection services at least two years ago. Participants were recruited through four non-government organizations across New South Wales that support vulnerable mothers; mothers were provided with information about the study by their support workers. Study 2 involved ten mothers who self-identified as managing to balance mothering with mental health recovery and were not involved with child protection services. Participants were recruited through newsletter advertisements of three community mental health non-government organizations across New South Wales. Eligibility criteria for the two original studies are presented in Table 1. Interviews across both studies were between 50 and 100 min long with an average of 70 min. Participants provided informed consent, either in writing or verbally on audiotape. Despite not being the focus of either study, the importance of partners to mothers’ experiences was striking in every interview.

**Table 1:** Eligibility criteria

Study 1 (*n* = 8)	Study 2 (*n* = 10)
Biological mother	Biological mother
Over 18 years of age	Over 18 years of age
Self-identifies as experiencing mental illness;	Self-identifies as experiencing mental illness;
Not currently under acute care (e.g., inpatient care) for mental Illness	Self-identifies as being able to balance mothering with mental health recovery
One or more children removed by child protection services more than 2 years prior to the interview.	Not currently involved with child protection or crisis services (including acute care).

### Interviews

Both original studies involved in-depth, semi-structured interviews. Interviews were conducted by a registered occupational therapist and post-graduate student (MM - Study 1) and a final year occupational therapy honours student (RA – Study 2) in 2013 and 2015 respectively. Both were female and both had experience in clinical interviewing and were trained and mentored by their supervisors (AH,NH,RM) in research interviewing. Neither had prior relationships with participants. Single interviews were conducted in private at a location chosen by participants: at a community venue; at the participants home; or by telephone.

Both studies used flexible interview guides to allow participants to describe and expand on what was most important to them. This allowed for a rich understanding of personal perceptions, meanings and experiences [34]. All interviews were audio-recorded and transcribed verbatim for detailed analysis and fieldnotes were taken to aid interpretation. In Study 1, interviews explored the impact of child removal on mothers’ lives and how they adapted to their new situations. In Study 2, interviews focused on understanding the strategies used by mothers to balance the demands of mothering with mental health recovery. Both sets of interviews included questions about the supports and barriers in women’s social networks. Further details about the original studies can be found in their respective publications [9, 37].

### Data analysis

Whilst a grounded theory cannot be developed using secondary data, techniques from grounded theory, specifically constant comparative analysis and memo-writing were used to analyze the data. These grounded theory techniques have been successfully used in previous secondary analysis studies [20, 39] and provide a systematic and well-established set of procedures for developing an explanation of how a phenomenon is experienced when there is limited understanding of a topic [40, 41]. Given that the two studies had different primary aims, used different qualitative approaches and involved asking participants different questions, the resulting narratives were also different. Grounded theory techniques were valuable as they enable the combination of different types of data and do not require a complete or comparable narrative from each individual [40].

All data relating to partners (including children’s fathers and mothers’ other intimate partners and ex-partners) were coded in detail, using line-by-line coding [41]. Each chunk of data was compared to other chunks of data to identify and name underlying concepts [42]. For example, the quotes “they said to me ‘had [partner] signed that piece of paper you would not have lost your children’” and “the official reason they took him [was the] risk of future psychological abuse from the step-father” were identified as indicating the same concept – the partner as ‘reason for child removal.’ As new data were coded, chunks of data were compared to existing substantive codes. If they did not fit, the code name was adjusted or a new code was generated [41]. Comparing codes to each other enabled them to be grouped into higher level conceptual categories [42]. For example, ‘reason for child removal’ and a number of other codes such as ‘restricting access to child’ were identified as ways in which partners’ disabled mothers’ opportunities to parent the way they wished and became the basis for the role category of ‘dismantler’. Comparison of codes to each other also allowed the relationships between them to be established [42]. This process, known as theoretical coding, resulted in the development of a framework to explain the roles played by partners in the mothering experiences of women living with mental illness.

Throughout the process, memos were created in a notebook to keep track of thoughts and ideas as they developed [41], providing an audit trail to enhance rigor. The first and second authors coded the first transcript independently and met to reach consensus around substantive codes. The first author coded the remaining transcripts, meeting regularly with other authors to discuss interpretations and coding in detail to enhance trustworthiness. The qualitative computer software QSR NVivo10 was used to manage and organize the data [43].

## Results

### Participants

Participants were a diverse group of mothers. Table 2 provides participant demographics split by study. The eight mothers who had at least one child removed by child protection services all attributed removal to perceived risk (rather than actual harm) to the child. While this sometimes related to the symptoms of mental illness, mothers more frequently described preceding issues with substance abuse and domestic violence perpetrated against them by a male partner. Four mothers reported having at least one child restored or awaiting restoration. Of the five mothers who had at least one child not in their care, two saw their children weekly, one saw her child bi-monthly, while two currently had no access to their children. Mothers discussed between one and three partners with an average of two partners, all of whom were male.

**Table 2:** Participant Characteristics

Characteristic	Study 1 (*N* = 8)	Study 2 (*N* = 10)
Mothers’ ages	Data not collected	30–55 years (mean = 42 years)
Children’s ages	1–22 years (mean = 8 years)	2–25 years (mean = 12 years)
Living with partner	3	6
Number of children	1–3 (mean = 1.75)	1–4 (mean = 1.9)
One or more children removed by child protection services	8	0
Current custody^a^		
Full time	3	7
Part time	0	4
Non-custodial	6	0
Experienced domestic violence	8	2
First diagnosis		
Prior to child’s birth	8	7
After child’s birth	0	3
Diagnoses^b^		
Depression	3	6
Bipolar disorder	3	2
Psychotic disorder	2	3
Anxiety disorder	1	3
Post-traumatic stress disorder	1	3

^a^ 1 mother from each study had different custody arrangements for different children

^b^ Mothers were not required to disclose a diagnosis. All but one did so, and almost half reported multiple diagnoses

To ensure confidentiality, pseudonyms unique to this article are used only for the first mention of each mother. Ethical approval was granted from the university’s Human Research Ethics Committee for each of the original studies and separately for the current secondary analysis study.

### Conceptual framework

**Fig. 1:**
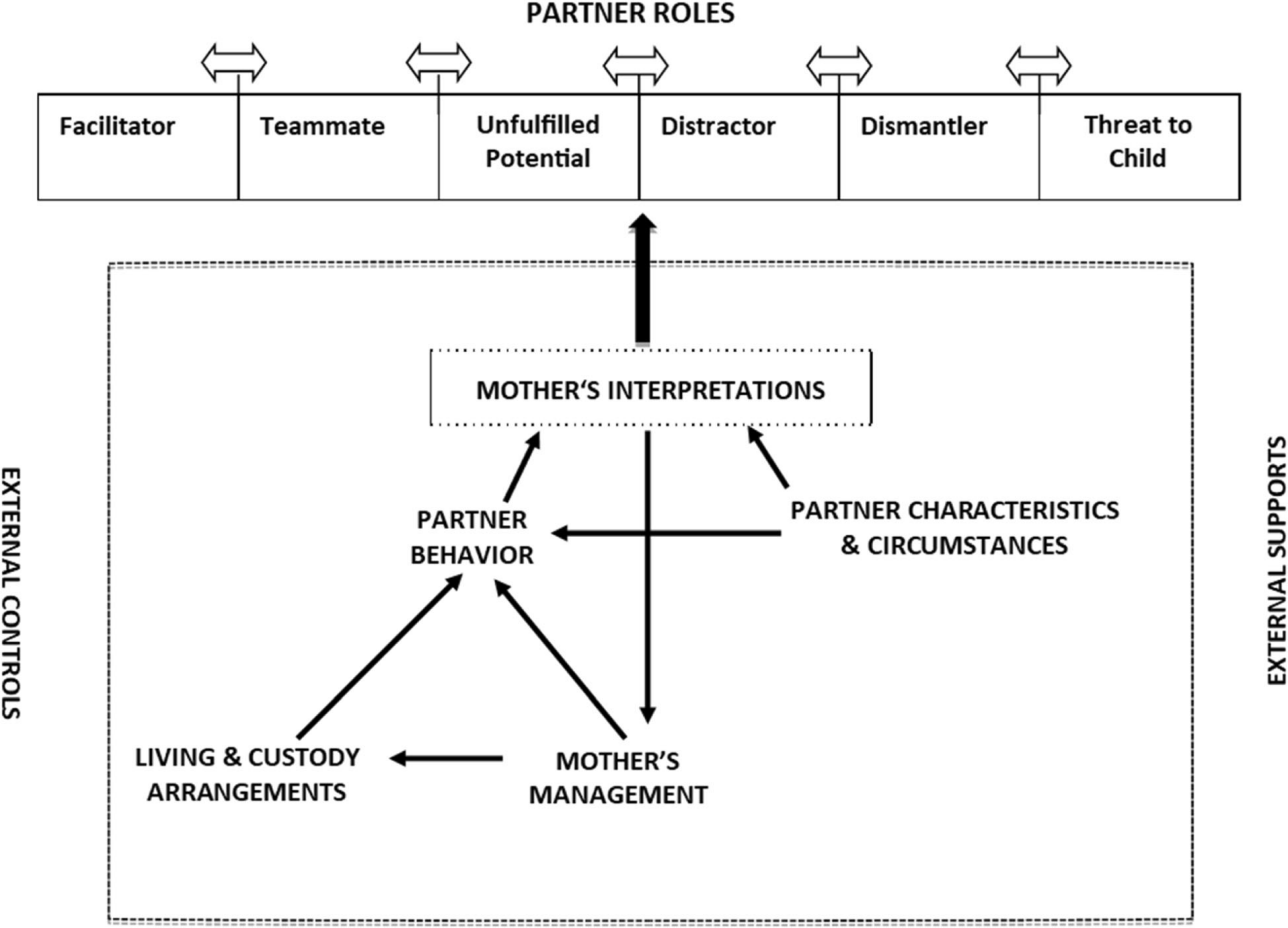
Roles of partners in the mothering experiences of women living with mental illness

The conceptual framework that explains the roles played by partners in the mothering experiences of women living with mental illness is depicted diagrammatically in Fig. 1 and described below.

Participants’ stories revealed six roles partners played in participants’ mothering experiences. These were facilitator, teammate, unfulfilled potential, distractor, dismantler and threat to child. Each partner discussed by participants played a unique combination of these roles. The two-headed arrows between the roles in Fig. 1 represent the potential of the different roles each partner played to expand, contract, shift and change over time.

The roles played by a partner at any time were shaped by the mother’s perceptions of both his behavior and his individual characteristics and circumstances. His behaviors, in turn were thought to be influenced by his characteristics and circumstances, the family’s living and custody arrangements, and how the mother herself actively managed his involvement. These interacting factors were also affected by controls and supports that were external to both mothers and their partners. The overall impact of a partner on a woman’s mothering experience depended on the mix of his roles and his proximity to the mother in time and space.

### Partner roles

The six roles partners played are described below. The numbers provided indicate the number of mothers in each study whose interviews contained data that contributed to each category, split by original study (in the form of *nstudy1 + nstudy2 = ntotal*). Because secondary data were used, interviews were not standardized, and partners were not the main focus of the interviews, it cannot be assumed that these numbers accurately represent the exact number of mothers in the sample who experienced each type of role.

#### Facilitator (*n* = 2 + 9 = 11)

Several mothers discussed partners who provided support that facilitated their ability to mother. For some, this support was critical to their parenting. Mandy stated, “if it wasn’t for [my child]’s dad I really don’t think I could’ve done this whole parenting gig.”

Partners facilitated mothering in diverse ways. Some provided support specifically focused on mothering. They did this by doing things like providing or paying for transport so that mothers could access their child, offering respite or ‘me time’, and giving helpful advice and perspectives. For instance, Colleen said, “I hadn’t realized that in my stress I was flustering my son. So it took my husband to politely point it out to me until I realized that in my anxiety, that I was causing him to get stressed.” Partners also provided more general support that was seen as helpful for mothering. This included financial support, advocating with health professionals, understanding, encouragement and motivation. As Janet explained: “he just sort of allows me to be where I am and doesn’t get up-tight if I am a bit low, or he is quite good at calming me if I am getting a little bit manic.”

#### Teammate (*n* = 4 + 9 = 13)

While the role of facilitator assisted a mother in her parenting, the role of teammate was identified when a partner himself performed parenting tasks, working alongside the mother to meet the needs of the child. For example, Monica described her partner “helping bath [my child], look after [my child], feed him.” Having a teammate (regardless of whether this was the child’s biological father) reduced the day to day burden of mothering tasks. Further, partners in some cases stepped up when the mother was unwell, for instance, Rowena said, “He had to take them home and feed them and put them to bed. So he had to take over the mothering role in a way.” In cases where the children were removed, teammates were involved in court cases, visited the children in foster care and suffered alongside mothers.

#### Unfulfilled potential (*n* = 8 + 10 = 18)

The role of unfulfilled potential represented the difference between the support a partner provided and what a mother needed or wanted from him. Even in the most supportive relationships, partners sometimes provided less support than needed or the wrong type of support. This was the case for Tracy’s current partner who, she said, “does try to help but [is] sometimes just off the mark.”

For others the unfulfilled potential was much greater. Some partners, like Rachel’s ex-partner, were simply absent, representing a complete lack of support: “He never wanted to be around … I never had financial aid from anybody, no one, nothing, so I was doing it on my own.” Other mothers described partners who spent little time at home or were present but disengaged, as Kathleen reported: “He was just floating around …. Not really part of things. Not really helpful or supportive or understanding of what was required.”

#### Distractor (*n* = 6 + 6 = 12)

Partners functioned as distractors when they took the attention, time and energy that mothers felt should have been focused on mothering their children. For a couple of partners, whose distracting role seemed minimal, this was just through annoying the mother with an excess of concern about her. Other partners were described as part of a chaotic lifestyle characterized by drugs, alcohol and partying. Louise recalled “I was more worried about what my partner was doing and smoking pot. Yeah, rather than enjoying the baby.”

Partners could also be a source of worry and concern. Mothers reported being distracted by their partners’ problems, such as health issues and unemployment as one mother described: “I realized that I was burning out again, trying to support my husband emotionally as well, and just, I wasn’t coping at all.” In some cases, the distraction was more sinister. Mothers’ capacity to focus on their mothering was compromised by the threat, fear, and results of domestic violence. Denise recalled, “It’s like I was only working at half capacity because half of me was always looking over my shoulder all the time.”

#### Dismantler (*n* = 7 + 4 = 11)

Several mothers described partners functioning as dismantlers, separating them from their children and their support networks, disempowering them and breaking down their ability to parent. Importantly, seven of the eight mothers whose children had been removed described partners as major contributors to the removal. One mother, for example, reported that child protection services “took [my child] saying there is a risk of future psychological abuse from the step-father.”

Where partners had custody, they sometimes made it hard for mothers to see their children. Helen’s ex-partner, for example, took her daughter to live in another state, limiting the frequency of visits. Partners could also diminish a mother’s parenting capacity through decreasing their confidence or ability to parent the way they felt best. For example, Julia described her parenting becoming more protective than she would have liked as a result of her ex-partner’s violence.

A less direct and often unintentional form of dismantling occurred when mothers were removed from their sources of support, such as family and friends. For example, Grace spoke about her partner being a barrier to support from her own parents, who disapproved of him: “It’s like they wanted to support me, but they wanted me to be away from my husband in order to support me.” Another mother described being separated from her supports because of her partner’s job: “When you move to another area, you don’t have your support structures.”

#### Threat to child (*n* = 6 + 2 = 8)

Several mothers described having had at least one partner who they believed posed a direct threat to their child’s well-being. Witnessing domestic violence was seen as traumatic for children, but children were also sometimes direct victims of the abuse: “The night terrors, they just continue for her, and I think her remembering him throwing her down and putting her hands behind her back and sitting on her and telling her how fat and stupid she is.” Threat could also be related to the partner neglecting the child or providing sub-optimal care. For example, one mother told how “the house she had lived in with my husband was totally unsuitable. … she wouldn’t eat and there were never hot meals cooked.” Mothers’ views of a partner as a threat to a child did not always coincide with the assessment of risk made by child protection services. For example, a couple of mothers saw child protection’s concerns about the child’s safety in relation to the partner as unreasonable, while in other cases, child protection services had supported fathers to gain custody when mothers saw them as a considerable risk to the child.

#### Multiple and shifting roles

**Fig. 2:**
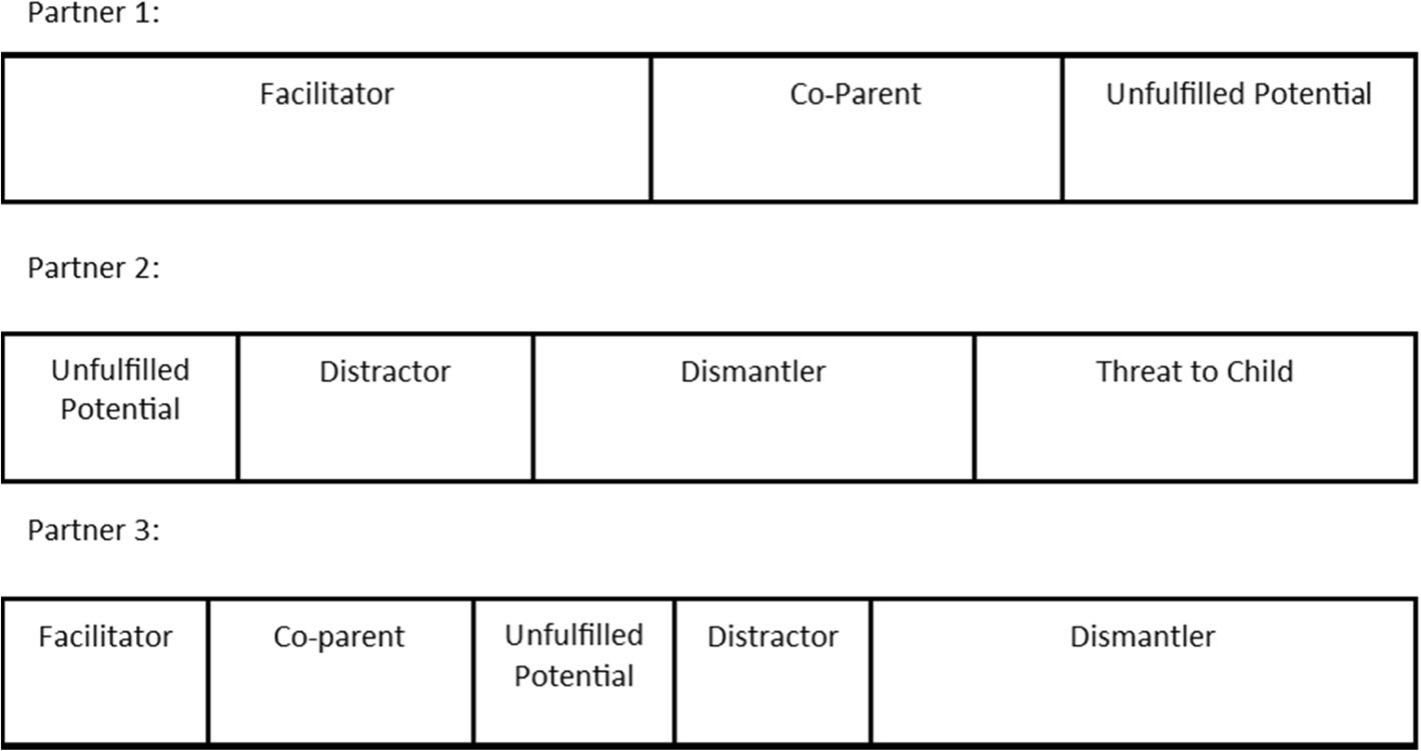
Examples of role combinations

Partners’ roles are shown in Fig. 1 as equal sections of a long rectangle. However, each partner to whom participants referred appeared to play a unique combination of these roles, with some roles dominant and others absent altogether. For example, Fig. 2 depicts an interpretation of the combination of roles played by three different partners of three different women. It is acknowledged that this is only the researchers’ interpretation from the available data and may not accurately reflect the individual women’s perceptions.

It is worth noting that positive and negative roles can occur together. For example, Courtney’s partner facilitated her mothering by providing financial support and acted as a teammate through hands-on childcare. However, his history of violence and refusal to sign a safety plan were grounds for removal, dismantling Courtney’s access to her child.

While role combinations were unique to each partner, differences were also apparent between the two data sets. The mothers from Study 1, whose children had been removed, were more likely to report partners playing substantial roles of distractor, dismantler and threat to the child. Mothers from Study 2, who were managing parenting well, were more likely to perceive partners as having significant facilitating and teammate roles. Mothers also tended to speak differently about current and former partners. While some ex-partners were described as supportive in some ways, unsurprisingly, they were more likely than current partners to be depicted in significant negative roles.

The roles were sometimes described as shifting over time and these changes often had a huge impact on mothers and their circumstances. For instance, although one partner had formerly dismantled the mother’s relationship with her family and children, he took on a teammate role when he came out of rehabilitation free of drugs: “we were able to successfully get our section 90 application through the court and win our daughter back.”

### What shapes partner roles?

#### Mothers’ interpretations

Partners’ roles in women’s lives were subjective, representing what partners were *to mothers*. While partners’ behavior and personal characteristics and circumstances influenced these roles, it was not only the observable factors, but how mothers subjectively interpreted these specific factors, that was important. For instance, some mothers were annoyed by behaviors others might find supportive, such as always wanting to discuss issues: “To me, I’ve found it a nuisance.” Interpretations in the face of seemingly similar situations differed across participants depending on their circumstances. For example, one mother saw her partner’s injury in a car accident as a stressor that distracted her from parenting, while another viewed her ex-partner’s illness as her “chance to pay him back” for his years of support of her. Similarly, a partner’s absence could be seen as unfulfilled potential (needed support not being provided), a distraction from parenting (missing the person), or the withdrawal of a previously dismantling or distracting influence.

#### Partner behavior

Clearly a partner’s behavior affected the roles he played. Sometimes partners, like Monica’s, reassured mothers, provided sound advice, and practical help: “he says ‘Monica come on let’s go see [your son]. Don’t feel bad about not seeing him, just get in the car and go see him.’” At the other end of the spectrum, one mother’s  ex-partner “would be coming around and threatening me, harassing me.” A single type of behavior could contribute to multiple roles. For example, domestic violence could be (depending on the mother’s perceptions) experienced simultaneously as a threat to the child, a dismantler and a distractor. Mothers believed that partner behaviors were influenced by a combination of his characteristics and circumstances, the living and custody arrangements under which they lived and her own management of the situation.

#### Partners’ personal characteristics and circumstances

Mothers attributed their partners’ behaviors to multiple personal and situational issues, such as their personality, emotions, family history, education and mental and physical health. For example, one mother attributed her partner’s violent behavior to his own struggles with alcoholism and ineffectively treated depression: “he was put on anti-depressants, but they weren’t working for him”. Another believed that her partner’s caring nature as well as his profession helped him to provide her with needed emotional support: “I’m married to a psychologist, I have to talk about it (laugh).” Mothers also talked about their partners’ behavior being influenced by their ability to cope with the mother’s illness. For example, one mother stated that her partner struggled emotionally because “it’s difficult sometimes to live with me.” Partners’ personal characteristics and circumstances also more directly influenced their roles. For example, where a mother saw the partner as being in need or in distress, this could be a source of stress and distraction for her. Similarly, a previous criminal record contributed to one partners’ dismantling role by being a consideration in the child’s removal.

#### Living and custody arrangements

Living and custody arrangements influenced partner behavior because they determined both a partner’s contact with the mother and children and his power to influence them for better or worse. Where partners were living in the same house with women and their children or had frequent contact, they were able to provide more hands-on support for both the child and the mother: “You get help with cooking and different, you know, daily chores, which I think is really important as well just to try and manage it.” However, cohabitating also provided a greater opportunity for violence or abuse: “he got out [of jail] he was back at my place and everything was fine for 6-8 months and then everything started going crazy again.” Similarly, partners who had been awarded custody were able to either support or disrupt the mother’s relationship with the child. For example, two mothers with children living with their fathers some distance away had different experiences. While one “barely got to see [my child] ‘cause I could never pay for a trip”, the other had a partner who facilitated her relationship with her children by paying for their visits.

#### Mother’s management

While the features described above shaped partners’ behavior, mothers were not passive and often acted to influence or manage their partners’ behavior. Sometimes they did this directly, for example by telling the partner what they needed or discouraging particular behavior. One mother, for example, had developed strategies to manage her partner’s support when it became too much: “I’ve had to learn to say I’m fine, I’m the same as what I was … ten minutes ago. Leave it at that. So you just have to shut him down.” At other times, mothers influenced partner behavior by altering their living or custody arrangements, such as leaving a partner or agreeing to shared custody. Important behavior changes were attributed to mothers’ actions. For example, one mother left her partner telling him “I’m through with you … you’ve lost your daughter, you’ve now lost your partner.” This action resulted in his addressing his substance issues and changing his behavior.

Mothers’ interpretations of partners’ behavior and situations shaped the management strategies they used. For example, Brooke reported putting up with her partner’s violence because she “thought that was the normal way” until a change of perception led to her ceasing that relationship.

#### External support and control

Each of the factors described above were impacted by supports and controls that came from outside mothers’ relationships with their partners. Supports included informal supports, like family and friends. For example, having family support could reduce mothers’ perception of unfulfilled support needs as Lorna described: “I rang my mum and said could she come, and I need you to come and look after the girls for a while.”

Formal support from parenting services, health services and other organizations was critical. Support and education for partners could change their behavior, as Patricia found after attended parenting classes with her partner: “Now we’re both singing from the same hymn sheet.” It was also thought to affect partners’ characteristics and circumstances, such as their mental health and ability to cope: “I just wish there was more supports in place for him as well. Someone he can talk to besides me.” Family services were also influential. For instance, one mother revealed how their situation worsened when child and family services were withdrawn from them: “We slipped under the radar, things were going from bad to worse um, violence was creeping back in, alcoholism was creeping back in.” Group or individual therapy helped mothers to perceive their partners’ behavior and circumstances in different ways. This sometimes led to active management to change situations, like addressing domestic violence, or altered roles due to changed perceptions. For example, one mother realized that her distracting worries about her partner were caused by her own insecurity: “I learnt that he kept coming back and he was willing to do everything, and I could trust him.”

The main external controls discussed by mothers in this study were child protection services and the courts. Together, they had two major impacts. First, for mothers whose children had been removed, or who had separated from their partners, they determined the custody and access arrangements for their children. This sometimes put partners in the position of being able to facilitate or impede mothers’ relationships with their children. For example, one mother whose ex-partner was awarded custody told how: “I was restricted to what food I could bring [to] what the father would let me. What I could let her drink, what I could say, what I couldn’t say, which was basically nothing.” Second, in some cases, fear of child protection services prevented mothers from managing situations with their partners as they might have liked. Several mothers reported feeling that they had to keep quiet about negative situations to avoid removal of their children, rather than seeking help to change them. One mother described it as being “damned if you do, damned if you don’t,” which in turn increased her partner’s ability to dismantle, distract and be a threat to her children.

## Discussion

To our knowledge this is the first study to specifically focus on exploring the roles of partners in the mothering experiences of women living with mental illness. It has identified six core roles partners may play, from mothers’ perspectives, and the complex factors that impact on these roles. The findings highlight three issues that are important for health and social service workers to consider when interacting with mothers living with mental illness. First, findings confirm the importance of partners in mothers’ experiences. Second, they suggest a more nuanced view of the roles of partners than is often assumed and reported in the literature. Third, findings highlight the place of mothers in determining or shaping partner roles, thus enhancing the possibility of positive change.

The importance of male partners to the wellbeing of children and mothers has been demonstrated in the general population (e.g., [44–46]). Further, there is increasing evidence of the value of parents working in partnership. The coparent relationship or parent alliance, is focused on child-rearing, is distinct from other aspects of the relationship, exists even after marital separation, and reflects parents’ ability to cooperate to best meet the child’s needs [47]. Cooperative co-parenting, characterized by parents supporting each other in child care and parenting decisions, has been shown to result in positive outcomes for mothers and children (e.g., [48–51]). Fathers’ support of mothers has been found to be important in establishing this cooperative coparenting relationship [13].

Because of the evidence of the importance of male partners’ roles, increasing emphasis has been given to “father inclusive practice” in parenting, child health and parent mental health interventions [52, 53]. For example, researchers in postpartum depression in particular are calling for partners to be involved in treatment given evidence that their support can both help the mother to recover and buffer the child and father against the negative impacts of maternal depression [54]. Yet there is still often a perception of the father as secondary in the child-rearing process and a consequent neglect, in research and practice, of their well-being and roles [55, 56]. Findings from the current study highlight the importance of the support provided by partners to mothers as well as the potentially detrimental impact of negative partner roles [30]. The findings are consistent with family systems theory [57, 58], in which the experiences and behaviors of each family member are understood to affect the functioning of the entire family. Following this theory, partners’ roles are likely to have a considerable impact on the relationships between mothers and their children, and on children themselves. The findings reinforce calls for services to involve partners in parenting support and maternal mental health [53]. Similarly, it should be acknowledged that partners’ wellbeing and ability to provide support can be negatively influenced by a mother’s mental illness [54], creating a vicious cycle. Supporting partners to cope, manage and recognize how their behaviors may be impacting on mothers is an investment that can maximize partners’ support of mothers who live with mental illness, positively influencing the entire family.

The roles identified in the current framework are consistent with partner behaviors reported in previous studies of mothering with mental illness more generally. For example, mothers have described their partners caring for children when they became unwell (teammate), being understanding and someone to rely on (facilitator), and depleting a mother’s energy (distractor) [7, 31]. This alignment with other literature supports the credibility of the proposed framework and suggests potential applicability beyond the current study participants. However, this study adds to the existing literature by providing a more nuanced view of partners’ roles. Previous literature has tended to conceptualize partners as black and white – either positive or negative. For example, where partners provided support for parenting or general support they were described as assets [7, 31]. On the other hand, where they were abusive or unsupportive, they were considered hindrances [10, 21, 22, 31]. The current study proposes a model in which partners can hold multiple roles concurrently, both positive and negative. The potential to play concurrent roles has been previously identified in research on the role of partners of mothers with intellectual disabilities which highlighted that some abusive men were also supportive [36]. Despite this emerging complexity, the partners of mothers with intellectual disability were still categorized as supportive or non-supportive [36]. The current findings suggest that from the perspectives of mothers living with mental illness, the role of their partners is more complex with partners identified as having the potential to play six different roles to varying degrees- some supportive, others hindering. This suggests that dichotomizing partners as either supportive or unsupportive/detrimental may not fully reflect the experience of mothers living with mental illness. In addition to this, the findings suggest that roles are not static as implied by past research. Rather, the proportions can shift over time. Viewing partners as more complex may open the way for services to facilitate this positive shift and work with partners’ strengths rather than simply dismissing them as ‘unsupportive’.

This study highlights the role of mothers themselves in determining and shaping their partner’s roles. The framework highlights that partners’ roles are not objective; rather they are a combination of partners’ behavior and mothers’ interpretations. Mothers also demonstrated the potential to actively shape situations. Some mothers brought about positive change through accessing parenting services or limited their partner’s ability to dismantle and distract by leaving them. This will be no surprise to family systems theorists, who see family systems as dynamic and each family member’s roles as a product of negotiations between family members [57]. Nevertheless, the active role of mothers has been largely ignored in previous literature in this area, with studies focused on how mothers felt or things that had happened to them [4]. Booth and Booth [36] identified that roles could shift for partners of women with intellectual disability, however usually in negative directions due to stressors such as child removal and loss of support. In contrast, the current study indicates that, for women living with mental illness, not only is positive change possible, but the women themselves often actively contribute to this positive change. In line with a family systems perspective, coparenting research indicates that the parenting relationship is not determined by fathers’ behavior only, but by mothers’ behavior as well [13], for example, through maternal gatekeeping [59]. Although cooperative coparenting may not be possible in some abusive relationships, it is seen as amenable to intervention for many parents [50]. While mothers’ ability to shape partners’ roles are clearly limited by circumstances such as legal issues and personal resources, acknowledging the agency of mothers reinforces the importance of supporting mothers to enhance positive change.

### Strengths & Limitations

The combined 18 interviews provided a substantial pool of qualitative data which contained rich descriptions relating to partners, enabling the development of an empirically based conceptual framework. Through the inclusion of data from two very different groups of mothers, the framework may be applicable to a wide range of mothers, from those managing their challenges well to those who have struggled, and from those who have full-time care of their children to those who are mothering as non-custodial parents.

However, there are several limitations inherent in secondary analysis, which should be considered in interpreting the findings. The original studies used different approaches and answered different questions; neither focused specifically on the roles of partners. It is therefore likely that women may not have described all relevant experiences and the data therefore may not paint a full picture for each individual. Further, there was no opportunity to extend the sample to ensure saturation of codes. Despite this the rich data available meant that the categories were well filled out. A strength of this secondary analysis is that it was conducted by the original researchers and was closely related to the original aims of the research, increasing the researchers’ understanding of contextual information and the trustworthiness of the interpretation [38, 60].

The study involved a small sample of women from New South Wales, Australia who had accessed mental health or parenting services for support. The women were all from English speaking backgrounds and described male partners only. Therefore, the applicability of results to mothers who are not in contact with services, have female or other gendered partners, or are from other cultural backgrounds should be considered with appropriate caution. The age range of participants’ children was broad and women discussed the impact of partners from early in their children’s lives to the current time. This meant that the analysis was based on a breadth of remembered experience rather than enabling a deeper exploration of issues that might be specific to mothers of children of particular ages.

## Conclusions

Given the lack of research to date, this secondary analysis is an important starting point for future research specifically focused on gaining an in-depth understanding of the complex roles of partners in the lives of mothers living with mental illness. Further research is needed to confirm, modify or add to the framework developed in this study. Research investigating partners’ own perspectives in understanding their roles and the supports they need is warranted in order to facilitate positive influences in the lives of mothers living with mental illness.

As part of the “external supports” in parents’ lives, health and social service providers are in a position to influence partners’ roles, support mothers to actively shape partners’ roles and, therefore, affect mothers’ experiences. The findings suggest that partners may be an under-recognized and under-supported social resource to assist mothering in the context of mental illness. To provide context sensitive health care to this group of mothers, health professionals need to acknowledge the specific importance of partners in a mother’s broader environment. This study provides a framework through which services can assist mothers to identify the roles that partners play, recognize the impact of these roles on their mothering and find ways to optimize their potential. Direct support for partners can be used to enhance their ability to support mothers.

In summary, this study suggests that partners are an important part of a mother’s world. Partners need to be acknowledged and included in service provision to facilitate what has the potential to be a huge natural support.

## Data Availability

The datasets analyzed during the current study are not publicly available as they consist of audio files and transcripts from in-depth interviews which, even with pseudonyms, might potentially allow individual participants to be identified.

## Abbreviations

n: the number of mothers (out of a total of 18) whose interviews contained data that contributed to each category
Partners: The fathers of women’s children and women’s other intimate partners or ex-partners
